# Improving the Decay Resistance of Wood through the Fixation of Different Nanoparticles Using Silica Aerogel

**DOI:** 10.3390/gels10040255

**Published:** 2024-04-10

**Authors:** Miklós Bak, Zsófia Plesér, Róbert Németh

**Affiliations:** Faculty of Wood Engineering and Creative Industries, University of Sopron, H9400 Sopron, Hungarynemeth.robert@uni-sopron.hu (R.N.)

**Keywords:** wood modification, decay resistance, silica aerogel, nanoparticle, fixation

## Abstract

Nowadays, the protection of wood is becoming more important with the increasing demand for durable wood, in addition to its limited accessibility. One possible way to increase the durability is the use of nanoparticles, which can be effective even with a low intake of active ingredients. However, avoiding their leaching is a challenge. A possible solution to leaching is the use of silica aerogel as a fixative. This study investigated the use of mesoporous silica aerogel against the leaching of different nanoparticles under laboratory conditions. Tests were performed involving beech (*Fagus sylvatica*) and Scots pine (*Pinus sylvestris*) sapwood, using *Trametes versicolor* as a white rot and *Coniophora puteana* as a brown rot fungus. The results show that the subsequent treatment of the wood with mesoporous silica aerogel effectively fixed the nanoparticles in wood. The durability of the samples without aerogel significantly decreased as a result of leaching, whereas the resistance of the samples treated with aerogel decreased only slightly. However, the silica aerogel modification itself caused the leaching of silver nanoparticles, which is a limitation in the use of this method for the fixation of nanoparticles.

## 1. Introduction

The lack of available European wood species with high natural durability requires extensive research on improving the durability of wood. Besides the common wood preservation methods, several wood modification processes have been developed during the last decades, and many processes are now used in the wood industry [1]. These processes, including the use of nanoparticles (NPs) and structures to improve the physical properties, mechanical properties or durability of wood is a current research topic [2,3,4].

The use of different metal nanoparticles provides high protection against different wood-decaying fungi, or even termites. Commonly investigated nanoparticles found to be effective in earlier studies are different formulations of copper, boron, silver, titanium, cerium and zinc [5,6,7,8,9,10]. Besides laboratory tests, field tests have proven the efficiency of NPs in wood protection. In some cases, they performed better than the conventional wood preservatives used as controls [11]. The biological resistance of wood might be improved against both decaying fungi and mold. It was also found that nano-copper, nano-zinc and nano-silver provide effective protection against the formulation of *Penicillium funiculosum* and *Aspergillus brasiliensis* [12].

A typical problem of wood preservation or modification methods is the fixation of the agents in wood which are used to improve the targeted wood properties. This phenomenon is present in NP treatments as well. In the available literature, there are data on low and high leaching rates regarding NP treatments. According to the results, NP formulations in combination with emulsifiers (e.g., acrylic emulsion) or surfactants are resistant to leaching [7,13,14,15,16,17,18].

Different inorganic and organic silicon formulations are often used for wood modification. Usually, improvements in hydrophobicity, dimensional stability, fire resistance and durability were reported [19,20]. Alkoxysilanes are frequently used for this purpose, as their use of hydrophobization agents have already been proven on various materials [21,22,23,24]. They are reported to improve the hydrophobicity and decay resistance of wood [25,26,27,28]. Tetraethoxysilane (TEOS) is one of the most commonly used alkoxysilanes, which is used to produce aerogel, in addition to its other uses [29,30,31].

Silica aerogels are low-density materials with a porous structure and high surface area. Their nanoscale structure consists of silica nanoparticles, forming an open porous 3D particle network. This hierarchical structure results in outstanding physical properties, such as high porosity (>90%), low flammability, low thermal conductivity and low bulk density (typically 0.05–0.3 g/cm^3^) [32]. Furthermore, the surface chemistry of silica aerogels can be modified to achieve superhydrophobic or oleophobic surfaces [33,34]. The combination of silica aerogels and cellulosic materials at the nano- and micro-scale is mainly used to synthesize high-performance insulating materials for the production of new-generation building solutions [35,36,37]. Another research direction is to improve the durability, mechanical properties, fire resistance or dimensional stability of wood, using silicon alkoxides and their derivatives as basic precursors of aerogels [4,30,38,39,40,41,42,43,44].

In a previous work, it had already been proven that silver, copper, copper–borate and zinc–borate NPs are effective against wood decay fungi [17]. However, the leaching resistance of these NPs was low. The objective of this study was to evaluate a post-treatment method which is used to fix the metal NPs in the wood cell walls in order to prevent their leaching. The efficiency of different nanoparticles in preventing the wood from decay by using low concentrations was already proven, but usually they exhibit low leaching resistance. Thus, there is a need for methods to eliminate this deficiency. Silica aerogel has a large specific surface area, coupled with a strong adsorption capacity [32], which is expected to prevent the metal nanoparticles from leaching. An in situ sol-gel synthesis of porous silica aerogel inside the cellular structure of wood was used for this purpose, as a simple and inexpensive method. The study investigated the effect of the combined treatment using metal NP impregnation, followed by the in situ silica aerogel formation, on the decay resistance and the leaching resistance.

## 2. Results and Discussion

### 2.1. Weight Percent Gain

Table 1 shows the weight percent gains (WPG) of the NP-treated samples (WPG_NP_), which only includes the NP uptake of the samples. As expected, the WPG shows low values for both wood species. The NP uptake was in proportion to the concentration of the used suspensions. Impregnations with nanosuspensions of the same concentration had the same impregnation efficiency (the same WPGs). The type of NP used did not affect the impregnation efficiency. The samples were divided into two groups, according to whether or not they were subjected to leaching during the decay test. There was no significant difference between the NP uptake of these two sample groups. However, there was a significant difference between the NP uptake of the different wood species. In addition to applying the same treatment parameters, a higher WPG was usually observed in Scots pine samples, typically 1.5–2 times higher. This result can be explained by the different density and anatomical structure of the two wood species, primarily by the different pore volumes [45,46].

The fixation of the NPs was carried out by applying silica aerogel, using the in situ sol-gel method. Table 2 shows the WPGs of the aerogel-treated samples (WPG_A_), which only includes the increase in weight of the samples after the aerogel formation, excluding the WPG values caused by the NP impregnation. The WPG in this case was independent from the type of the NP pretreatment, considering the NP type and concentration. Thus, the NP pretreatment did not influence the impregnability of the samples. However, there was a significant difference between the WPG_A_ values of beech and Scots pine. WPG_A_ was nearly 1.5 times higher for Scots pine. The average WPG_A_ values varied between 12.55 and 19.26%, depending on wood species, which is a relatively low value among silica-based wood modifications ranging from 20 to 60% [47].

### 2.2. Scanning Electron Microscopy (SEM) Imaging and Energy-Dispersive X-ray Spectroscopy (EDX) Analysis

**Figure 1:**
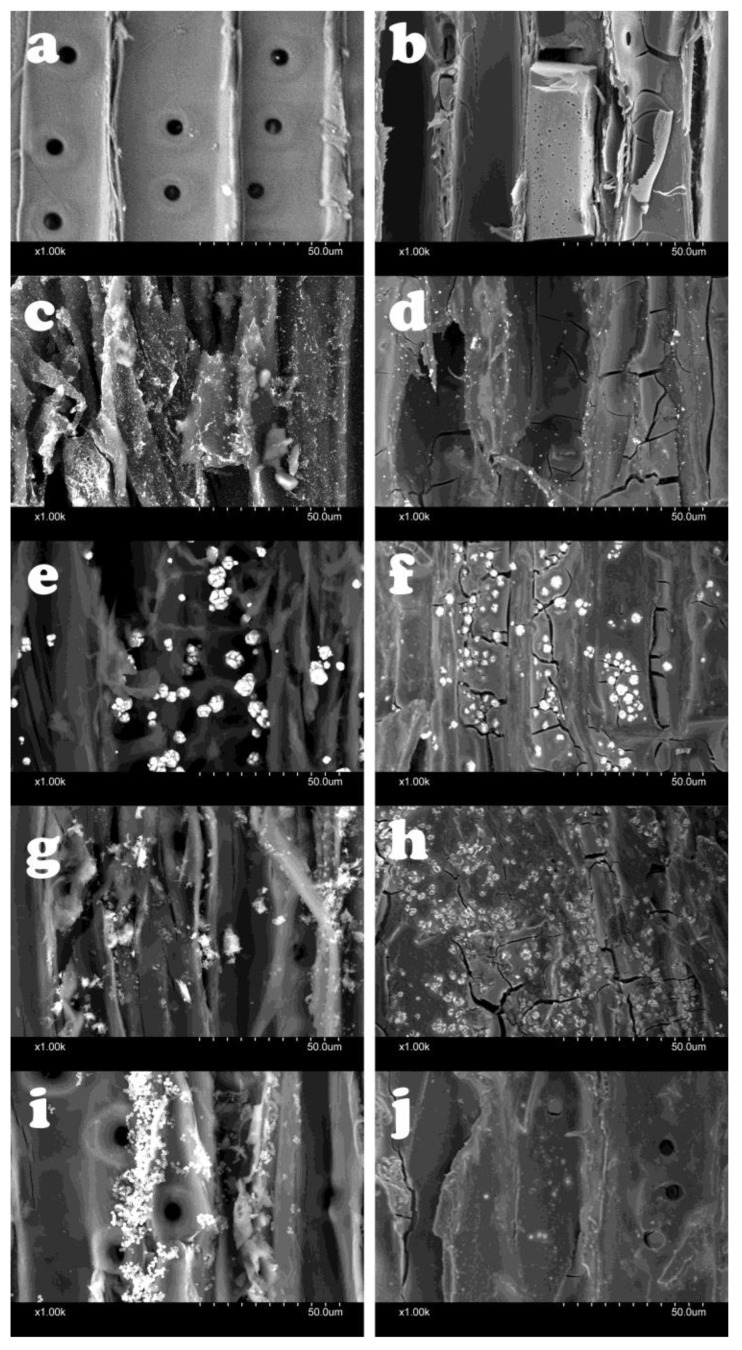
SEM images of the untreated (**a**), silica aerogel-modified (**b**), silver nanoparticle-impregnated (**c**), silver nanoparticle-impregnated and silica aerogel-fixed (**d**), copper nanoparticle-impregnated (**e**), copper nanoparticle-impregnated and silica aerogel-fixed (**f**), copper–borate nanoparticle-impregnated (**g**), copper–borate nanoparticle-impregnated and silica aerogel-fixed (**h**), zinc–borate nanoparticle-impregnated (**i**), zinc–borate nanoparticle-impregnated and silica aerogel-fixed (**j**) Scots pine sapwood.

When compared to the untreated material (Figure 1a), pure silica aerogel was visible in the SEM images as a coating on the cell walls and larger deposits, which clogged the cell lumens entirely (Figure 1b). Several cracks were visible in the structure, which was the result of shrinkage of the aerogel during the drying and curing process [48]. This result indicates that the low amount of silica precursor used was enough to cover the cell walls entirely and, thus, fix the metal nanoparticles properly. The metal NPs were also clearly visible in the SEM images (Figure 1c,e,g,i). In addition to the presence of single NPs on the cell wall surface, agglomerations were also visible. They were especially conspicuous in copper, where the original NP size should have been the smallest (2–4 nm) among the investigated NPs, but the copper NP agglomerations were much larger than this size. The agglomeration of the NPs decreased the specific surface area, which might have led to a reduction in their efficiency as protective agents against wood-decaying fungi. After the silica aerogel formation on the cell walls, the NPs were still visible, embedded in the aerogel (Figure 1d,f,h,j). However, silver NPs were present in a visibly lower amount. Also, zinc–borate NPs were less visible after the aerogel fixation.

**Figure 2:**
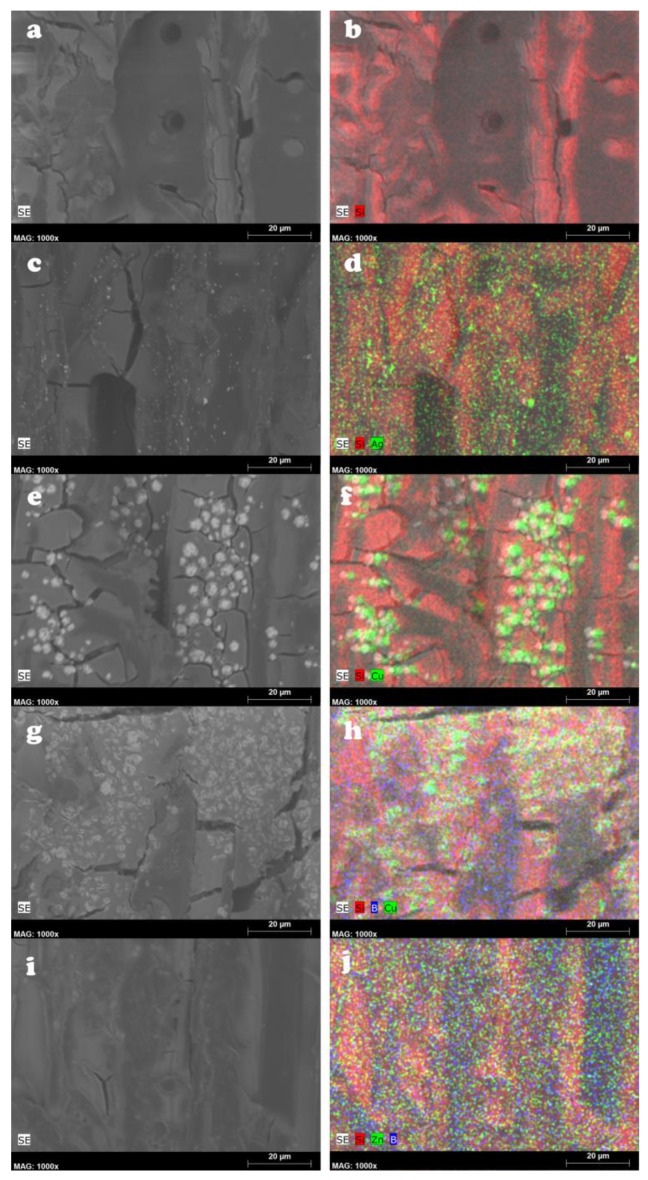
SEM images of the silica aerogel-modified (**a**), silver nanoparticle-impregnated and silica aerogel-fixed (**c**), copper nanoparticle-impregnated and silica aerogel-fixed (**e**), copper–borate nanoparticle-impregnated and silica aerogel-fixed (**g**), zinc–borate nanoparticle-impregnated and silica aerogel-fixed (**i**) Scots pine sapwood and the EDX elemental maps of the same surfaces (**b**,**d**,**f**,**h**,**j**).

Parallel to the SEM imaging, EDX analysis of the samples was carried out, to obtain more detailed information about the presence of metal NPs and aerogel. These results coincided with the data from SEM imaging, since they showed the presence of NPs on the cell wall surface, beside the agglomerations of both the NPs and aerogel in the cell lumens (Figure 2a–j). This result indicates that a considerable amount of metal NPs remained on the cell wall surfaces; besides that, some leaching of the metal NPs was expected during the aerogel fixation process. The embedded metal NPs were clearly visible in the elemental maps as well.

### 2.3. Decay Test

Considering all parameters tested, it can be concluded that the NP treatments provided somewhat higher protection against the white rot *Trametes versicolor*, compared to the brown rot *Coniophora puteana* (Figure 1 and Figure 2). In a previous study [17], the results showed that using 0.5, 1.0 and 2.0 m/m% concentrations of copper NPs did not provide adequate protection against *Coniophora puteana* or *Trametes versicolor*, and silver NPs did not protect against *Coniophora puteana*. Therefore, a higher NP concentration was applied here for these treatments than previously (5 and 7 m/m%). In this way, adequate efficiency was achieved against the investigated fungal species. The mass loss (ML) was kept below 3%, which is the efficiency limit determined by the relevant European standard [49]. WPG_NP_ (Table 1) shows the higher amount of silver and copper NPs in wood, with proportionally higher values compared to the copper–borate and zinc–borate NPs (0.5, 1.0 and 2 m/m%). According to the previous study, silver NPs provided adequate protection against white rot fungus, even after the leaching test, at lower NP concentrations (0.5, 1.0 and 2 m/m%). Therefore, it was not tested against white rot fungus in this work. This finding, regarding silver NPs, is in accordance with earlier studies, where it was found that silver NPs provided good protection against white rot fungi, but could only suppress the degradation caused by brown rot fungi [50,51].

The results show that, against *Coniophora puteana*, only copper NPs could not keep the ML below 3%. According to earlier studies, copper NPs provided higher durability compared to using bulk copper, because of its higher reactivity originating from its increased specific surface area, the lower viscosity of the NP-based formulations and the so called “reservoir-effect” that provides extended protection for wood [5,52,53,54,55]. Since the copper NPs were not able to provide high levels of protection to the wood, even with the significantly increased concentration used here, it can be stated that their use for this purpose is not recommended. Brown rot fungi are considered to be copper-tolerant [56]. Usually, higher copper concentrations are necessary to inhibit the growth of brown rot fungi, compared to white rot fungi [57]. Beside the copper tolerance of the test fungi, the strong inclination of copper NPs to form large agglomerations (Figure 1e,f) was another reason for showing lower efficiency against *Coniophora puteana*. On the other hand, all applied NPs and concentrations were effective against *Trametes versicolor*.

The higher tolerance of brown rot fungi against copper and silver NPs is related to the ability of such fungi to influence the pH of their close environment. The reduction in the pH is promoted by the production of different chelating compounds (e.g., oxalic acid) during the entire decay process [58,59]. This process reduces the toxicity of metals to the fungi, since these chelates are insoluble and are, accordingly, biologically unavailable forms of oxalate [60,61]. However, depending on the fungus species, other mechanisms might work in the background as well, like metal ions binding to cell surfaces, the development of efflux pumps, biomethylation or the removal of metal ions by precipitating [62].

When using silica aerogel to prevent leaching, the fungal resistance showed differences in several cases. Unfortunately, silver NPs lost their effectiveness against the brown rot fungus. Depending on the wood species and the concentration of silver NPs, the MLs, which were originally between 1.21 and 2.58%, increased to 19.89–29.70% after the aerogel modification, which was close to the ML of the control specimens. This result is supported by the SEM images (Figure 1c,d), in which a considerably lower amount of silver NPs was visible after the aerogel fixation process. This contradictory result did not occur to such an extent with the other investigated NPs, although, in most cases, a slight but significant increase was detectable at ML. However, this difference was, at most, 3%, as the initial value of 1.04% increased to 4.01% after the aerogel modification with zinc–borate 0.5% NP treatment for Scots pine. These results show that the in situ aerogel formation method used for fixation caused some leaching of the NPs originally introduced into the wood. This phenomenon was more pronounced with the silver NPs, so this method cannot be used for the fixation of silver NPs in wood. However, the procedure seems suitable for this purpose for other NPs. Contrary to these results, a previous study [51] reported a better leaching resistance of silver NPs than of copper NPs; additionally, silver NPs only reduced fungal activity in the case of brown rot fungus and inhibited it in the case of white rot fungus. An important difference is that a usual leaching process uses distilled water as leaching agent, while, during aerogel modification, a mixture of water and organic solvent (ethanol) is used. The presence of ethanol seemed to promote the leaching of silver NPs.

Due to the ML increase caused by the aerogel modification, the NPs that originally showed a ML of less than 3% at all concentrations and against both fungal species did not stay below this limit. The copper NPs remained below 3% ML only against the white rot fungus. In CuB and ZnB NPs, the use of a concentration of at least 2% resulted in a ML below 3% against both white rot and brown rot fungi after the aerogel modification of wood.

**Figure 3:**
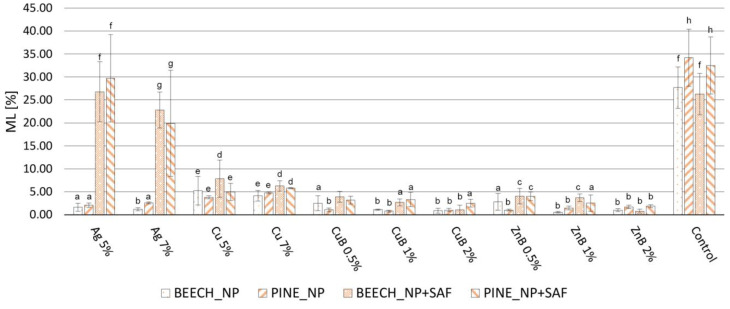
ML values of the nanoparticle-impregnated (NP), nanoparticle-impregnated and silica aerogel-fixed (NP + SAF) and control samples of beech and Scots pine sapwood, caused by the brown rot fungus *Coniophora puteana*. Different superscript letters indicate a significant difference between the WPG values of nanosuspension-impregnated samples at *p* < 0.05 level. SD denotes standard deviation.

**Figure 4:**
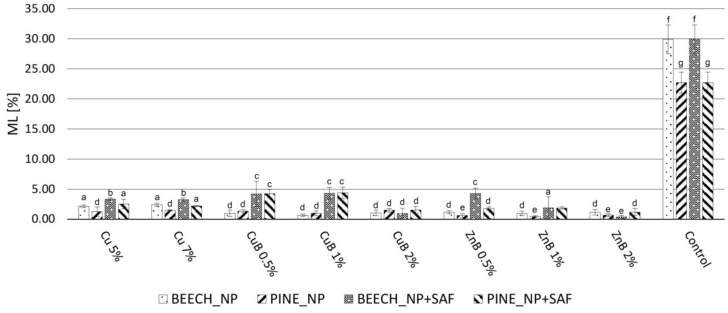
ML values of the nanoparticle-impregnated (NP), nanoparticle-impregnated and silica aerogel-fixed (NP + SAF) and control samples of beech and Scots pine sapwood, caused by the white rot fungus *Trametes versicolor*. Different superscript letters indicate a significant difference between the WPG values of nanosuspension-impregnated samples at *p* < 0.05 level. SD denotes standard deviation.

As a result of leaching, the degree of fungal decomposition increased uniformly in the case of specimens without aerogel fixation (NP) (Figure 3 and Figure 4). In specimens treated with copper NPs, the fungal resistance decreased to the smallest extent; however, their protection against brown rot fungus was not high initially, so only a small level of effectiveness was detected after leaching. The specimens treated with silver NPs lost their durability against the brown rot fungus after leaching, which shows that they were easily removed from the wood with water. The samples treated with CuB and ZnB completely lost their resistance against brown rot and white rot fungi, so it was especially valid for them that they were easily removed from wood during leaching with water [17,63].

The silica aerogel used as fixing agent to solve this problem significantly improved the durability of the specimens, even though this treatment itself caused some NP leaching (Figure 3 and Figure 4). Based on the fungal decay results, the silver NPs were already leached from wood during the aerogel modification used for fixation. As a result, silver NP + SAF specimens did not effectively resist the applied brown rot fungus, even after the leaching test. Specimens treated with copper NP + SAF showed only slightly higher ML values after leaching, compared to the non-leached ones. However, they only could keep the ML under 3% when tested against white rot. The ML value was around 10% for both NP concentrations, which means they had better durability compared to untreated specimens, but it was still not a highly effective protection. Thus, the use of copper NPs is not recommended even with aerogel fixation, which is in contrast with the findings of an earlier study [51], wherein copper NPs were found to be effective against the brown rot fungus *Poria placenta*.

**Figure 5:**
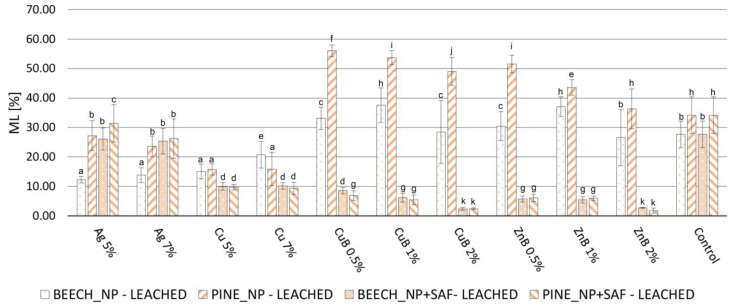
ML values of the nanoparticle-impregnated (NP), nanoparticle-impregnated and silica aerogel-fixed (NP + SAF) and control samples of beech and Scots pine sapwood after leaching process caused by the brown rot fungus *Coniophora puteana*. Different superscript letters indicate a significant difference between the WPG values of nanosuspension-impregnated samples at *p* < 0.05 level. SD denotes standard deviation.

**Figure 6:**
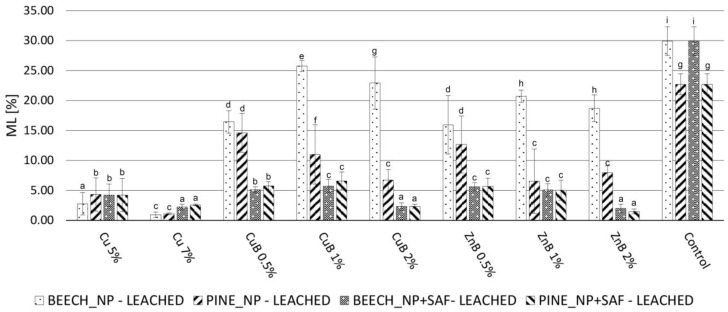
ML values of the nanoparticle-impregnated (NP), nanoparticle-impregnated and silica aerogel-fixed (NP + SAF) and control samples of beech and Scots pine sapwood after leaching process caused by the white rot fungus *Trametes versicolor*. Different superscript letters indicate a significant difference between the WPG values of nanosuspension-impregnated samples at *p* < 0.05 level. SD denotes standard deviation.

In contrast, the samples treated with CuB NP + SAF and ZnB NP + SAF showed significant resistance after fixation with aerogel. This result shows that fixation with aerogel effectively prevented the leaching of CuB and ZnB NPs from the wood. Only 5–10% ML was observed after aerogel fixation, even in samples treated with 0.5% and 1.0% NP suspensions. This was still not considered full protection, but it was already a significant step forward in terms of durability. With the CuB NP + SAF and ZnB NP + SAF treatments, the highest protection was achieved by using a 2% NP concentration, which resulted in an ML of less than 3% for both tested fungal species. Overall, depending on the test parameters (wood species, NP concentration, fungus species), a slight 1–5% higher ML was observed for the NP + SAF specimens after leaching, compared to the unleached specimens (Figure 5 and Figure 6). The effective prevention of NP leaching was also supported by the result that specimens treated only with silica aerogel did not mitigate the ML values caused by the fungi.

The increase in durability shown for the NP + SAF specimens can be explained by several factors. One of the primary effects of the silica aerogel modification is that it clogs the cell lumens and cell wall micropores [48], which might theoretically inhibit the penetration of fungal hyphae. On the other hand, the ML observed in the specimens treated only with aerogel was the same as that of the untreated specimens (Figure 3 and Figure 4), and thus, such an effect could not prevail in this case, which was in line with previous findings [29]. The effectiveness of the treatment was due to the fact that the silica aerogel was able to effectively prevent the leaching of NPs that were stuck to the cell wall surfaces and entered the micropores of the cell. Another reason was that the aerogel turned the wood hydrophobic, which further reduced the penetration of liquid water into the lumens of wood, thereby indirectly preventing leaching. The interaction of the wood components and the aerogel, which lead to the improved hydrophobicity and the lower moisture uptake, was proven using the Fourier Transform Infrared Spectroscopy (FT-IR) method in a previous study [48].

## 3. Conclusions

This study investigated the use of silica aerogel to prevent the leaching of different metal nanoparticles. The efficiency was tested directly through laboratory-scale fungal tests using the white rot *Trametes versicolor* and the brown rot *Coniophora puteana*. The efficacy of the nanoparticles was verified, with the exception of copper nanoparticles. In this case, despite the higher concentrations used, it was not possible to keep the mass loss below 3%. Therefore, the use of the tested copper nanoparticles is not appropriate for the high-level protection of wood. The use of silica aerogel modification process step to prevent leaching also caused some leaching of the nanoparticles. This leaching effect was only remarkable in the case of silver nanoparticles. Although they provided a high degree of protection against the fungal species included in the study, the method used to fix them caused their leaching, canceling their protection effect. Copper–borate and zinc–borate nanoparticles showed low leaching resistance without fixation, but the use of silica aerogel modification provided a high fixation rate for them, according to the decay tests. The mass losses caused by the test fungi were kept under 3% when 2% concentrations of copper–borate and zinc–borate nanoparticles were used.

## 4. Materials and Methods

### 4.1. Nanoparticle Treatment of the Samples

Four different nanoparticles were used in this study: silver (Ag), copper (Cu), copper–borate (CuB) and zinc–borate (ZnB). The nanosuspensions were used to impregnate the samples directly. The preparation of the nanosuspensions was carried out as described in a previous study [17]. Briefly, aqueous precursor solutions of the metal salts served as the base for NP formation, while different reducing agents were used to obtain metal NPs (Table 3). NPs were prepared at the highest concentration used for the tests and diluted further to lower concentrations by adding distilled water.

Wood samples of beech (*Fagus sylvatica*) and Scots pine sapwood (*Pinus sylvestris*) were cut into blocks of 15 mm × 25 mm × 50 mm (radial × tangential × longitudinal). The initial moisture content of the wood was 12 ± 2%.

Impregnation of all samples was carried out according to the relevant European standard [49]. Samples with a known oven-dry mass (m_0-NP_) were impregnated with the nano-suspensions. The impregnation process consisted of a 15 min vacuum at 0.7 kPa, followed by the introduction of a NP suspension to the vessel and the maintenance of the specimens at atmospheric pressure in the suspension for 2 h. Following the impregnation, the samples were dried at 103 °C in an oven and weighed afterwards to obtain the oven-dry mass after impregnation (m_1−NP_). According to these data, the weight percent gain (WPG_NP_) (m/m%) was calculated according to Equation (1), as follows:

(1) $${WPG}_{NP}=\frac{m_{1-NP}-m_{0-NP}}{m_{0-NP}}\cdot 100\left[\%\right]$$

### 4.2. Fixation of the Nanoparticles in Wood

The fixation of the nanoparticles was carried out by a porous SiO_2_ aerogel, using the in situ sol-gel method [48]. For aerogel preparation, a molar ratio of 1:5:8, regarding tetraethyl orthosilicate (TEOS): ethanol (ET): distilled water (H_2_O), was used. The hydrolysis was promoted by adding hydrochloric acid (HCL) dropwise, until the pH reached the value of 3. The mixture was stirred at 500 rpm at 50 °C for 60 min; it also turned clear from its initially opaque color.

Samples with a known oven-dry mass (m_1−NP_) were impregnated by the prepared silica-sol. The treatment consisted of a 60min vacuum phase under 10 kPa pressure at 25 °C and an impregnation step at atmospheric pressure, keeping the samples in the sol for 2 h. The impregnated specimens were then placed in an oven at 60 °C for 24 h, followed by another 24 h at 105 °C to age the gel, until SiO_2_ aerogel formed in situ inside the wood structure. Afterwards, samples were weighed to get the oven-dry mass after impregnation (m_1−A_). According to these data, the weight percent gain (WPG_A_) (m/m%) was calculated according to Equation (2), as follows:

(2) $${WPG}_{A}=\frac{m_{1-A}-m_{1-NP}}{m_{1-NP}}\cdot 100\left[\%\right]$$

After the impregnation process, the samples were climatized at 20 °C and a 65% relative humidity.

### 4.3. Scanning Electron Microscopy (SEM) and Energy-Dispersive X-ray Spectroscopy (EDX) Analysis

The structure of the metal NPs and silica aerogel in the structure of wood were investigated with a Hitachi S3400N scanning electron microscope. An accelerating voltage of 10 kV was used. The surfaces were coated with a sputter-coater prior to the imaging. We cut 5 × 5 × 5 mm samples from the middle part of the treated material. Longitudinal (radial or tangential) sections were used for this purpose. The elemental composition was determined by elemental mapping analysis using an energy-dispersive X-ray spectrometer, combined with SEM (Bruker XFlash Detector 5010, Billerica, MA, USA). The accelerating voltage was 10 kV. The element mapping was performed on a selected area of 70 × 100 µm with a spatial resolution of 0.1 µm.

### 4.4. Decay Test

The decay test was performed on the samples according to the relevant European standard [49]. The test fungi for this study were the brown rot fungus *Coniophora puteana* and the white rot fungus *Trametes versicolor*. The conditions during the test were a 23 °C temperature and 75% relative humidity.

The specimens were divided into the following three groups:

- Treated test specimens: impregnated specimens subjected to attack by the wood-destroying fungi. Five treated test specimens (NP-treated, aerogel-treated, and NP + aerogel-treated) were used for each NP concentration, timber species and fungus species.
- Treated and leached test specimens: impregnated specimens subjected to attack by the wood-destroying fungi, after a leaching procedure that was performed according to the relevant European standard [64]. Five treated test specimens (NP-treated, aerogel-treated, and NP + aerogel-treated) were used for each NP concentration, wood species and fungus species.
- Untreated test specimens: non-impregnated test specimens of the same wood species as those of the treated test specimens. They were placed in culture vessels next to the treated specimens and treated and leached specimens as a control.

One inoculated culture vessel contained one treated specimen, one treated and leached specimen, and one untreated test specimen. After the introduction of the test specimens, the culture vessels were placed in a climate chamber at a constant temperature of 23 °C and relative humidity of 75% for 16 weeks. Following incubation, the specimens were removed from the culture vessels, cleaned from the mycelium, oven-dried (103 °C ± 2 °C) and weighed (*m*_2_). The mass loss was calculated based on the oven-dry weight of the samples before incubation (*m*_0_), according to Equation (3), as follows:

(3) $$ML=\frac{m_{0}-m_{2}}{m_{0}}\times 100\;[\%]$$

### 4.5. Statistical Analysis of the Results

The distribution normality of the data was verified and statistical significance tests of analysis of variance (ANOVA) and Fischer least significant difference (LSD)-test, *p* < 0.05) were conducted for the effect of the treatment on the mass loss, using the software Statistica 10.0 (Statsoft, Palo Alto, CA, USA).

## Figures and Tables

**Table 1:** Weight percent gain of beech and Scots pine as a result of different nanosuspension impregnations (WPG_NP_).

Treatment Type	WPG_NP_ (%)							
	Beech				Pine			
	Normal		For Leaching		Normal		For Leaching	
	Average	SD	Average	SD	Average	SD	Average	SD
Ag 5% (m/m)	4.04 ^a^	0.33	3.53 ^a^	0.18	6.53 ^f^	0.29	6.54 ^f^	0.27
Ag 7% (m/m)	5.05 ^b^	0.40	5.07 ^b^	0.15	9.47 ^g^	0.68	9.16 ^g^	0.68
Cu 5% (m/m)	3.76 ^a^	0.36	3.41 ^a^	0.09	6.74 ^f^	0.39	6.41 ^f^	0.20
Cu 7% (m/m)	5.53 ^b^	0.55	5.00 ^b^	0.19	9.35 ^g^	0.80	9.07 ^g^	0.48
CuB 0.5% (m/m)	0.42 ^c^	0.03	0.39 ^c^	0.02	0.68 ^h^	0.04	0.65 ^h^	0.03
CuB 1% (m/m)	0.79 ^d^	0.08	0.73 ^d^	0.02	1.28 ^i^	0.06	1.29 ^i^	0.06
CuB 2% (m/m)	1.63 ^e^	0.15	1.48 ^e^	0.04	2.69 ^j^	0.21	2.51 ^j^	0.16
ZnB 0.5% (m/m)	0.38 ^c^	0.03	0.41 ^c^	0.01	0.68 ^h^	0.03	0.64 ^h^	0.03
ZnB 1% (m/m)	0.78 ^d^	0.05	0.73 ^d^	0.02	1.39 ^i^	0.12	1.32 ^i^	0.05
ZnB 2% (m/m)	1.66 ^e^	0.08	1.50 ^e^	0.03	2.68 ^j^	0.21	2.52 ^j^	0.09

Different superscript letters indicate a significant difference between the WPG of nanosuspension-impregnated samples at the *p* < 0.05 level. SD denotes standard deviation. Ag, Cu, CuB and ZnB denote silver, copper, copper–borate and zinc–borate nanosuspensions, respectively.

**Table 2:** Weight percent gain of beech and Scots pine as a result of silica aerogel modification (WPG_A_).

Treatment Type	WPG_A_ (%)							
	Beech				Pine			
	Normal		For Leaching		Normal		For Leaching	
	Average	SD	Average	SD	Average	SD	Average	SD
Ag-A 5% (m/m)	14.80 ^a^	1.64	12.31 ^a^	1.55	18.95 ^b^	1.09	16.74 ^b^	0.50
Ag-A 7% (m/m)	13.71 ^a^	1.97	11.93 ^a^	1.69	18.92 ^b^	1.81	18.21 ^b^	2.02
Cu-A 5% (m/m)	14.22 ^a^	0.79	12.28 ^a^	1.07	17.87 ^b^	2.20	17.86 ^b^	1.77
Cu-A 7% (m/m)	12.34 ^a^	2.02	12.42 ^a^	1.36	19.26 ^b^	1.13	18.78 ^b^	1.28
CuB-A 0.5% (m/m)	14.14 ^a^	1.58	13.90 ^a^	1.62	17.65 ^b^	1.20	18.15 ^b^	2.33
CuB-A 1% (m/m)	14.01 ^a^	1.13	12.71 ^a^	1.32	19.17 ^b^	2.49	18.58 ^b^	2.37
CuB-A 2% (m/m)	12.55 ^a^	1.59	14.77 ^a^	2.21	17.58 ^b^	2.04	18.60 ^b^	1.83
ZnB-A 0.5% (m/m)	13.42 ^a^	1.19	13.48 ^a^	1.12	18.32 ^b^	2.59	15.97 ^b^	2.82
ZnB-A 1% (m/m)	14.52 ^a^	1.53	13.64 ^a^	0.74	18.23 ^b^	1.52	17.82 ^b^	1.44
ZnB-A 2% (m/m)	14.78 ^a^	1.91	12.30 ^a^	1.56	18.96 ^b^	1.80	17.74 ^b^	1.70

Different superscript letters indicate a significant difference between the WPG of silica aerogel-modified samples at *p* < 0.05 level. SD denotes standard deviation. Ag, Cu, CuB and ZnB denote silver, copper, copper–borate and zinc–borate nanosuspensions, respectively.

**Table 3:** Details of the NP preparation methods.

Nanoparticle	Precursor (Supplier)	Reducing Agent (Supplier)	Protecting Agent (Supplier)	Average NP Size	NP Conc. (m/m%)
Silver	Silver nitrate ^a^	L-ascorbic acid ^b^	Polyvinyl- pyrrolidone ^c^	80–100 nm	5%
					7%
Copper	Copper (II) nitrate—anhydrous ^d^	L-ascorbic acid ^b^	Polyvinyl- pyrrolidone ^c^	2–4 nm	5%
					7%
Copper–borate	Copper (II) nitrate—anhydrous ^d^	Borax decahydrate ^e^	-	100 nm	0.5%
					1%
					2%
Zinc–borate	Zinc nitrate hexahydrate ^e^	Borax decahydrate ^e^	-	100–200 nm	0.5%
					1%
					2%

^a^ Scharlab, Debrecen, Hungary; ^b^ Sigma-Aldrich, St. Louis, USA; ^c^ VWR Chemicals, Radnor, USA; ^d^ Acros Organics, Geel, Belgium; ^e^ Acros Organics, Geel, Belgium.

## Data Availability

Dataset available on request from the authors.
